# Determining the recurrence rate of premature ventricular complexes and idiopathic ventricular tachycardia after radiofrequency catheter ablation with the help of designing a machine-learning model

**DOI:** 10.1016/j.reth.2024.03.001

**Published:** 2024-03-10

**Authors:** Entezar Mehrabi Nasab, Saeed Sadeghian, Ali Vasheghani Farahani, Ahmad Yamini Sharif, Farzad Masoud Kabir, Houshang Bavanpour Karvane, Ahora Zahedi, Ali Bozorgi

**Affiliations:** a Department of Cardiology, School of Medicine, Tehran Heart Center, Tehran University of Medical Sciences, Tehran, Iran; b Department of Cardiology, School of Medicine, Valiasr Hospital, Zanjan University of Medical Sciences, Zanjan, Iran; c Tehran Heart Center, Tehran University of Medical Sciences, Tehran, Iran; d Department of Artificial Intelligence in Medical Sciences, Faculty of Advanced Technologies in Medicine, Iran University of Medical Sciences, Tehran, Iran

**Keywords:** Artificial intelligence, PVC, RF ablation, Machine learning

## Abstract

Ventricular arrhythmias increase cardiovascular morbidity and mortality. Recurrent PVCs and IVT are generally considered benign in the absence of structural heart abnormalities. Artificial intelligence is a rapidly growing field. In recent years, medical professionals have shown great interest in the potential use of ML, an integral part of AI, in various disciplines, including diagnostic applications, decision-making, prognostic stratification, and solving complex pathophysiological aspects of diseases from these data at extraordinary complexity, scale, and acquisition rate. The aim of this study was to design an ML model to predict the probability of PVC and IVT recurrence after RF ablation.

Data of patients were collected and manipulated using traditional analysis and various artificial intelligence models, namely MLP, Gradient Boosting Machines, Random Forest, and Logistic Regression.

Hypertension, male sex, and the use of non-irrigate catheters were associated with less freedom from arrhythmia. All these results were obtained through traditional analytic methods, and according to AI, none of the variables had a clear effect on the recurrence of arrhythmia.

Each AI model presents unique strengths and weaknesses, and further optimization and fine-tuning of these models are necessary to increase their clinical utility. By expanding the dataset, improved predictions can be fostered to ultimately increase the clinical utility of AI in predicting PVC erosion outcomes.

## 1 Introduction

VAs increase cardiovascular morbidity and mortality [1]. In the absence of structural heart abnormalities, recurrent PVCs and IVT are generally considered benign. However, these arrhythmias often cause chest discomfort, which affects a person's quality of life and daily activity and also can lead to systolic dysfunction. However, in patients with PVCs-induced cardiomyopathy, RFA success often restores LV function gradually [[2], [3], [4], [5]]. Conversely, PVC and recurrent VT are common in patients with non-ischemic and ischemic cardiomyopathies. Early stages of DCM can be associated with life-threatening VAs [6]. Moreover, DCM can cause structural, electrical, and electromechanical remodeling, and this remodeling can further foster complex arrhythmias. The likelihood of successful VA ablation is significantly higher in patients with ICM (56–77%) than in those with DCM (38–67%). The IVTs are defined as arrhythmias that originate from ventricles but are not associated with any identifiable structural problem in the heart [1]. It is believed that IVT is caused by either fascicular bundle re-entry or stimulated focal activity. It is of crucial importance to precisely determine the mapping of these arrhythmias so that subsequent ablation interventions can be planned accurately, which is often curative. IVT is a large group of VA that includes different types: mitral annular VT, tricuspid annular ventricular tachycardia, ventricular outflow tract tachycardia, interfascicular ventricular tachycardia, and papillary muscle ventricular tachycardia. Although these arrhythmias show a benign nature, symptoms are frequently observed in affected individuals, requiring treatments such as usually prolonged therapeutic interventions with AADs. However, these interventions may fail to achieve clinical remission, and compliance to this type of treatment is often limited due to side effects or contraindications, where RFA can provide an effective alternative [7,8]. In patients suffering from PVCs and IVT, immediate alleviation following RFA can reach as high as 84%, showing a considerable rate of success. This promising value may reduce to 71% in the follow-up if no AADs are used; however, the use of AADs may increase the rate of success to 85%. Also, the success rate is influenced by the type of PVC and IVT. Patients with diseases originated from RVOT are more likely to enjoy remission, but individuals with epicardial and multifocal PVCs have been predicted to have a higher chance for failure. The factors known to have a negative impact on PCV are male gender, symptomatic disease, and epicardial disease origin. These factors have been suggested to increase disease burden and the risk of PVC-induced cardiomyopathy. On the other hand, recurrent PVCs and PVCs of RVOT origin have been reported to effectively resolve following RFA, rendering a plausibly safe procedure in these patients with a low rate of adverse effects [9,10]. Although RFA is considered to be an effective therapeutic intervention in RVOT-originated PVCs, this procedure is less effective in patients with PVCs of papillary muscles, a condition with a high recurrence rate, demanding the more extensive delivery of radiofrequency energy and longer operation time [11,12]. The high success rate of RFA at medium and short-term follow-up is well documented, but success in long-term follow-up is still uncertain [13].

Medical scientists are becoming more and more interested in ML, a subset of artificial intelligence. Modern medicine is witnessing an unprecedented increase in access to biological and also clinical data. ML sensors are envisioned to support medical decision-making and are expected to comprise an integral part of diagnostic and prognostic modalities in the future. ML can also help better understand the complicated pathophysiology of diseases with extraordinary complexity, scale, and acquisition rate. In comparing AI with human intelligence, it is necessary to mention that human intelligence can perform observation-based analyses, judgments, and decision-making. On the other side, AI works based on prespecified rules and procedures already fed into a computer. AI is based on two main ideas: 1- Providing thinking processes through machines and 2-study of human thinking processes.

Simultaneously with the growing demand to improve medical services in 1970, AI brought about a tremendous change in medical knowledge, which led to suggestions for designing computer programs to help doctors diagnose and treat diseases. Among the capabilities and applications of artificial intelligence in medicine, the following can be mentioned: A) Identification of storage and retrieval of information in the database, B) Developing disease treatment and control plans, E) Predicting the outbreaks of diseases, C) Interpretation of medical photographs, and D) Identification and diagnosis of diseases. Various types of ML can be classified as; 1) the type of learning and 2) type of task. Tasks of the ML are usually categorized based on whether the expected responses are available for the training of input data. Where objectives are a projection of unseen data and available for input data, the task is referred to as supervised learning. On the other hand, in unsupervised learning, target data are unavailable. The main objective here is to use appropriate transformation to seek suitable data representation and better data visualization. For this purpose, relatively fewer and more simplified constructs are utilized. In this regard, hybrid approaches such as semi-supervised learning may be employed, offering partially labeled training data, and the supervised task is mainly guided by unlabeled data. Another ML approach, called reinforcement learning, uses trial and error patterns for selecting tasks with the highest rewards, where labeling can be bypassed (a process that is central for supervised learning), and in fact, the reward function is responsible for guiding the system through its environment [14]. Regarding the types of tasks performed by ML, regression, classification, dimensionality reduction, and clustering are considered the key elements. Supervised learning is intertwined with classification tasks, where each input data is assigned to a limited number of discrete categories. Similarly, the spectrum of supervised learning also engulfs regression tasks by extending the classification problem by predicting ≥1 continuous variables. One should make a clear distinction between the predictive function of statistical regression (i.e., estimation of associations between exposure and outcome variables) and ML regression. Where unlabeled input training data are used in ML, one goal may be identifying datasets with similarities to each other (i.e. clustering) or reducing the number of dimensions of the data (e.g., for better 2D or 3D visualization of data for extracting discrete data features) [[14], [15], [16]].

The applications of ML are increasing in studying cardiac arrhythmias and electrophysiological features (ion channels, electrical currents, etc.). It seems that the use of ML in modern medicine can be very useful and therefore, we decided to design the present study with the aim of designing a ML model to predict the risk of PVC and IVT recurrence after RF ablation. Accurate predictions on the possibility of recurrence before performing any invasive procedure can give the cardiologist a wider view on planning the treatment process and scheduling follow-up monitoring.

## 2 Methods

### 2.1 Data collection

Patients over 18 years of age with PVC and IVT who were referred to Tehran's Heart Center Hospital between 2021 and 2022 and underwent catheter ablation were candidates to be included in this study. The sample size was determined as 520 people. Exclusion criteria were: 1) diagnosis of ischemic cardiomyopathy, 2) not revisiting for follow-up after catheter ablation, 3) diagnosis of congenital heart diseases, and 4) lack of consent to cooperate. Patient information was obtained by reviewing registered electronic files in the hospital's database. Primary information and clinical data were checked by two cardiologists to ensure accuracy. A final decision on the patient's eligibility for enrolment in the study was made after reaching a consensus between the two specialists.

This study conformed to the TRIPOD and all relevant protocols and was approved by the hospital's ethics committee. We followed the principles of the Declaration of Helsinki. Prior to any procedure (obtaining information, therapeutic interventions, etc.), informed written consent was obtained from all patients. First, the patient was contacted by phone, and after providing necessary explanations, informed consent was obtained from the patients. Basic information of the patients included a history of hypertension, age, smoking habits, sex, history of diabetes, hyperlipidemia, coronary heart disease, use of AADs, family history of diseases, medical history, alcohol consumption, addiction to psychoactive drugs, physical examination, echocardiogram indicators, laboratory data, patient information at discharge, and follow-up records. All these data were obtained by reviewing the medical records available in the data management system. Only the data of eligible patients were gathered. The final data were categorized and prepared for use in the ML algorithmalgorithm.

### 2.2 Variables and outcomes used in models

For this study, different prediction models were used, as mentioned below. Demographic variables included gender and age. Also, underlying diseases and risk factors included diabetes, blood pressure, hypertension, dyslipidemia, history of CABG, family history of heart disease, previous history of PCI, smoking, opium use, previous coronary artery disease, and congenital heart disease. Also, Holter burden, Morphology-match, Earliest Signal, Catheter, procedure time, and fluoroscopy time variables were used to design the model. The main outcome was the recurrence of PVC and VT.

### 2.3 Algorithm design

This article delves into the outcomes of various artificial intelligence models employed for this purpose, namely MLP, Gradient Boosting Machines, Random Forest, and Logistic Regression. LR algorithm belongs to the family of supervised ML models. It is also considered a discriminant model, meaning that it tries to distinguish between classes (or categories). A set of independent variables is used by LR to estimate the probability of the occurrence of an event. Where probability is the outcome, the dependent variable ranges from 0 to 1. Random forest algorithm is used to build an explainable model of the ML. Random forest is an ensemble algorithm based on DT. Bagging is the main idea of Random forest; and all independent DTs in the forest contribute to the simultaneous selection of features in the Random forest model (a process that conforms to majority subordination principles). After the integration of all DTs, Random forest can deliver a better performance than each individual DT, while avoiding over-differentiation. An optimized distributed gradient boosting library, XGBoost algorithm is regarded as a highly efficient, portable and flexible platform, employing ML algorithms under the Gradient Boosting framework. With high accuracy, the XGBoost provides a parallel tree boost (also known as GBDT, GBM) to solve dataset problems.

### 2.4 Divided into two groups, test/train, and selection of variables

In order to validate the prediction models, the population of this study was randomly divided into the training group (70% of the patients) and the test group (30% of the sample). Also, the best features were chosen by a feature selection algorithm on the training data, and the random forest model's superior features on the train data were obtained using k-fold cross-validation (k = 5). The model is adjusted employing a stronger predictor if a strong correlation is found between the two clinically related variables (based on Pearson's correlation coefficient). The models are designed once with the selected variables and once without them.

### 2.5 Oversampling and scaling

Imbalanced data must be dealt with in ML, and one strategy to balance the sample training data is to utilize the artificial minority sampling method (SMOTE). Since the test data points must remain obscure and unmodified, the test/train split was initially performed. Synthetic data related to the minority group (here, those without recurrence), were generated using the SMOTE technique so that an equal number of outcomes is achieved. Data scaling for each feature was finally performed using a standard scaler to eliminate mean and scaling to unit variance to develop prediction models.

### 2.6 Model development

Prediction models were developed employing a number of ML methods [1]: LR [2], Random forest, and [3] XGB. All models were designed using k-fold cross-validation (k = 5). We managed to enhance the accuracy of prediction models by parameter adjustment using the grid search method. The training and testing of each model were performed to predict PVC and VT recurrence.

### 2.7 Evaluating of model performance

The indicators of [1] prediction accuracy (accuracy) [2]; specificity [3]; sensitivity; and [4] the area under the receiver operating characteristic curve (ROC-AUC) score plotting the true positive versus false positive rate were used to evaluate the performance of ML methods. The model's performance is best predicted by AUC as an independent measure of the threshold and a main comparative index. The values of AUC of ≥0.9, 0.8–0.9, 0.7–0.8, 0.6–0.7, and <0.6 indicate outstanding, excellent, acceptable/fair, poor, and no discrimination, respectively. A threshold of 0.5 (50%) determines the cutoff for assigning a class label based on a predicted probability. This prediction cut-off was then adjusted using k-fold cross-validation (k = 5) to determine sensitivity and specificity and address the highly unbalanced outcome and low mortality rate.

### 2.8 Statistical analysis

For data description, SD or percentage was used, and comparative tests (Pearson's correlation, chi-square test, and Fisher's exact test) were used for the analysis of categorical variables. Continuous variables were analyzed by the independent sample *t*-test. All tests were interpreted at a two-sided p-value of <0.05. Model development and analyses were conducted in Python (3.10). XGBoost was developed using the XGBoost (1.6.0) Python library, and LR and Random forest models were implemented using the scikit-learn (1.0.2) library.

## 3 Results

### 3.1 Patient population

The mean age of the patients (n = 520) was 58 (18–79) years, and men constituted 59.3% of the participants (Table 1). Arrhythmia burden was 18.4% (7.4.-42.5) based on the data obtained from 48-h PVC rhythm monitoring Holter. The PVC index origin was distributed as follows: n = 197 (37.9) for LVOT; n = 76 (14.6) for non-LVOT; n = 185 (35.5) for RVOT; and n = 62 [12] for non- RVOT. The participants’ data have been summarized in Table 1.

**Table 1:** Recurrence characteristics in the patients.

Characteristic	Overall, N = 520	No reccurence, N = 361	reccurence, N = 159	p-value^2^
Mapping_3D				>0.999
NAVX/PRECISION	93.8%	93.3%	94.9%	
CARTO	6.2%	6.7%	5.1%	
Catheter				0.044
non-irrigated	11.9%	14.2%	6.8%	
irrigated	88.1%	85.8%	93.2%	
gender				0.006
Female	44.6%	38.1%	59.3%	
Male	55.4%	61.9%	40.7%	
DM	9.8%	9.0%	11.9%	0.532
HTN	27.5%	22.4%	39.0%	0.017
DLP	17.6%	19.4%	13.6%	0.326
CS	7.3%	9.0%	3.4%	0.234
Opium	4.7%	5.2%	3.4%	0.725
FH	3.1%	3.0%	3.4%	>0.999
CABG	7.3%	8.2%	5.1%	0.557
PCI	8.8%	9.7%	6.8%	0.509
LVEF				0.463
Median (IQR)	50.0 (40.0, 55.0)	50.0 (40.0, 55.0)	47.0 (40.0, 55.0)	

### 3.2 Procedural data

Among the 520 registered patients, ablation was performed in 458 patients using irrigated catheter ablation, while the rest were managed with non-irrigated catheters. Based on the machine learning model, no significant difference was reported between the type of catheter regarding the rate of recurrence. However, traditional analysis revealed that the rate of recurrence was significantly lower when irrigated catheters were used (P = 0.044). The total procedure time and fluoroscopy time were 70 ± 15.5 and 20 ± 8.3 min in those who relapsed and 68.9 ± 13.6 and 8.1 ± 7.8 min in people who did not have recurrence. No significant results were obtained in the approach using artificial intelligence. In traditional analysis, it was seen that the duration of the procedure did not influence the probability of recurrence and the average time.

### 3.3 Study outcomes

**Fig. 1:**
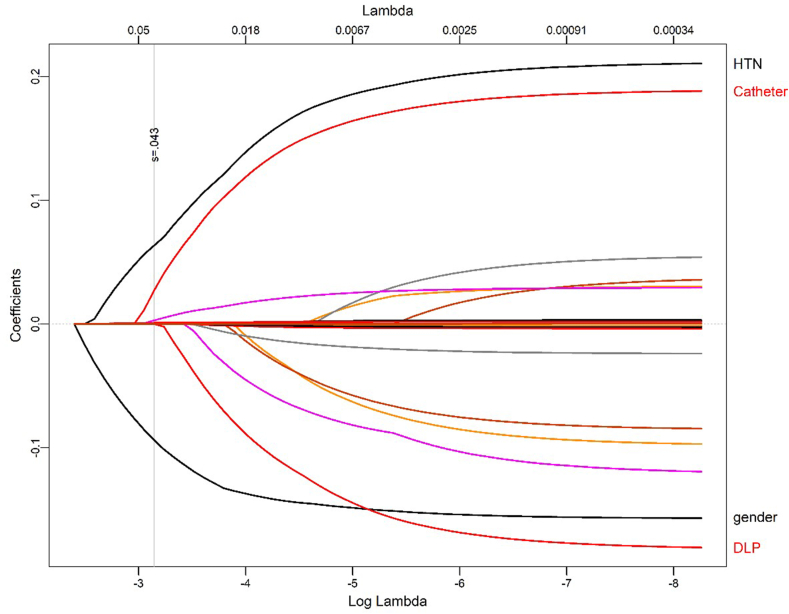
Selection of the best predictors for arrhythmia recurrence based on the LASSO method.

The average follow-up of the study was 6–12 months, and most patients experienced a remarkable reduction in PVC burden (a mean reduction percentage from the baseline: 89.1%, p < 0.05), and 361 (69.4%) patients remained arrhythmia-free. Also, 48-h PVC burden was reduced significantly regardless of the location of PVC. Hypertension, male gender, and use of non-irrigate catheters were associated with the risk of arrhythmia recurrence both in univariate and multivariate analyses (Table 2 and Fig. 1). All these results were obtained through traditional analytic methods. Using artificial intelligence, none of these variables had a clear relationship with the recurrence of arrhythmia.

**Table 2:** Multivariable logistic regression for selecting the best arrhythmia recurrence predictors.

Characteristic	OR^a^	95% CI^a^	p-value
Catheter			0.044
irrigated non-	–	–	
irrigated	2.71	0.91, 10.2	
Gender			
Female	–	–	
Male	0.42	0.22, 0.78	0.007
HTN	2.10	1.05, 4.23	0.036

a OR = Odds Ratio, CI = Confidence Interval.

### 3.4 Machine learning

a) MLP: The MLP model demonstrated reasonable precision in predicting arrhythmia recurrence (0.59), with a notable recall for the absence of recurrence (0.87). However, the model's performance diminished in the case of true recurrence (0.12). The overall accuracy of 0.56 suggested room for improvement, as indicated by convergence warning during optimization.
b) Gradient Boosting Machines: Similar to MLP, Gradient Boosting Machines showed promise in predicting non-recurrence (precision of 0.83) but struggled with true positive identification (recall of 0.19). The model achieved an overall accuracy of 0.56, emphasizing the need for refining its ability to detect arrhythmia recurrence.
c) Random Forest: The Random Forest model exhibited a high precision for non-recurrence (0.96) but faced challenges in correctly identifying true recurrences (recall of 0.06). The model's accuracy was obtained as 0.59, indicating its high predictive potential, yet its low recall demanded enhancing its predictive capabilities.
d) Logistic Regression: Logistic Regression offered a balance between precision and recall for both recurrence and non-recurrence. With a precision of 0.60 for non-recurrence and 0.72 for recurrence, the model delivered an overall accuracy of 0.59. However, recall accuracy for recurrence (0.12) suggested highlighted the need for improvement in identifying true positive cases.

## 4 Discussion

PVCs and IVT are usually found in patients with no evidence of structural heart diseases. However, some of these patients may develop underlying cardiomyopathy. A high daily load of paroxysmal VT or PVCs may cause heart dilation and cardiac systolic dysfunction(i.e., PVC-induced cardiomyopathy). A study reported that in elderly people with no evidence of cardiac abnormalities, a lower left ventricular EF was noted to correlate with the baseline PVC/VT burden [17]. In cases with IVA, when pharmaceutical therapy is rendered ineffective, CA provides a viable therapeutic option with class I indication [[18], [19], [20]]. It is beneficial to have an accurate prediction of the effectiveness of this procedure to design a personalized therapeutic protocol for the patient with a broader perspective.

AI is ubiquitous in today's world, and we encounter it on a daily basis. As a branch of AI, ML assigns functions such as learning, interpretation, and generation of ideas to machines that work based on expanded data sources. Cardiovascular medicine and especially electrophysiology are increasingly becoming the subject of ML; however, the path toward the clinical application of ML still faces numerous hurdles. ML is rapidly gaining interest in the interpretation of automated ECG parameters, but these applications are expected to disseminate in various disciplines such as understanding the pathophysiology and electrophysiology mechanisms of arrhythmias and electrical functional patterns of the heart [14]. This topic has been reviewed by several studies, affirming the rapid growth of research in this field, especially on future perspectives and applications, as well as hurdles and challenges [[21], [22], [23]]. In this regard, ML algorithms have been employed to interpret pulse and ECG irregularities and assist medical professionals in reaching accurate and reliable diagnoses. For example, in the Apple Heart Study [24], a smart-watch app equipped with an ML algorithm was employed to detect AF, offering a positive predictive value of 0.84, supporting the applicability of AI applications in understanding cardiac electrophysiology parameters [25,26] and assisting cardiologists in making timely and reliable decisions that can improve cardiovascular care. In a study, Yokokawa et al. used ML to localize scar-related VT exit from 12-lead pacing ECGs during ablation velocity mapping. A SVM was trained on digital velocity map ECGs with pacing locations aiming to detect the site of VT ablation based on VT ECG to provide a reliable and targeted guide for subsequent ablation [27]. For ML algorithms to become proficient predictors of the electrophysiology of cardiac diseases and arrhythmias, we need to gather several combinations of data sources to mimic complex and integrated biological processes at different levels. Since there is no need for manual feature engineering, DL offers a suitable tool in order to analyze complex raw datasets. The interpretability of DL can be boosted by the translation of millions of parameters in an NN into an interpretable reason for each decision (e.g., bolding selected parts of medical images, identifying ECG parameters with the greatest impact on classification, etc.). In a study by Hemalatha and colleagues, they used ML to more accurately and correctly distinguish VA from other arrhythmias [28]. In another study, researchers were able to distinguish between left and right ventricular VT with the help of ML. The use of AI techniques both makes clinical decision-making easier and increases the accuracy of the localization of VTs [29]. In the last decade, RFA has become a widespread treatment for VAs. But despite enormous progress in technologies and techniques, patients are still at significant risk of recurrence and mortality after VAs ablation. Significant efforts have been dedicated to defining the determinants of poor prognosis in these patients [[30], [31], [32], [33], [34], [35]]. However, we have not succeeded yet in developing a pre-RFA model to accurately estimate the risk of VT recurrence and mortality.

Yibo and his colleagues were able to predict the final recurrence rate in patients with AF before ablation with the help of an ML model [36]. We tried to use artificial intelligence to predict the possibility of arrhythmia recurrence before performing radiofrequency ablation. In the present study, we followed up on the patients who underwent RF catheter-based ablation due to IVT or PVCs between 6 and 12 months. The purpose of this study was to determine the main risk factors of disease recurrence and to design an AI algorithm to predict the probability of recurrence. Traditional data analysis demonstrated that several main factors were involved in increasing the risk of recurrence, including the type of catheter, gender, and history of HTN. We observed that the use of irrigated catheters was associated with a lower recurrence rate compared to non-irrigated catheters, and this difference was statistically significant. This result was logical because as far as we know, irrigated catheters affect a wider area and are applied more effectively. Further, it was found that HTN was an independent predictor of disease recurrence after successful ablation. In HTN, tissue remodeling at the molecular level in the heart may predispose to the recurrence of arrhythmia. Hemodynamic changes, atrial and ventricular structural remodeling and fibrosis, and a proarrhythmogenic electrophysiological phenotype of the hypertrophied left ventricle, as well as prolonged QTc are believed to increase the likelihood of developing HTN arrhythmogenesis [37]. Left ventricular hypertrophy is known as the main cause of VA and SCD in patients with hypertension, and it also plays a role in the recurrence of arrhythmia after its successful treatment [38]. Therefore, careful attention to the complete control of HTN and the discovery of other mechanisms through which HTN increases the incidence of IVT and PVCs can be an effective solution to finding strategies to prevent arrhythmia recurrence.

Gender was also an effective predictive factor for arrhythmia recurrence. The incidence of VA is generally higher in men, and we observed that not only the incidence of VA was higher in men, but also men were more likely to experience disease recurrence after RF ablation. Unfortunately, the results obtained from AI analysis were inconclusive, and it was not possible to design an algorithm to predict the probability of recurrence.

For designing efficient ML medical algorithms, it is important to deeply divulge and resolve the shortcomings of this technology [16]. The validation and effective learning of ML algorithms demands gigantic data sources, which is currently a noteworthy challenge of using ML in the clinical setting because clinical data are not openly available and shared across medical centers, thus democratizing data access. Public use of ML is of great importance to address the fear of using personal health data. In this study, we collected information from 520 patients during the years 2021–2022, but it seems that this sample size was not enough to design such an algorithm. Soalgorithm. So, it may be necessary to use data from other centers to be able to design effective algorithms based on the data of a much larger sample size. The solution to this problem is data sharing, especially for clinical trial data, which demands expanding safe and secure infrastructure for data storage and analysis. Also, issues such as intellectual property and privacy problems should be appropriately addressed before data sharing can happen. Another limitation is that ML algorithms can only learn patterns based on data-specific training on, so data restricted to a specific population cannot be applicable to design all-inclusive ML algorithms applicable on a global scale, presenting a major source of distrust in ML. The data used in this study were from a third referral center where all procedures were performed by a few very experienced operators, so our results may not reflect the results obtained by other less experienced operators. Second, our follow-up results obtained by 48-h Holter monitoring might not be able to estimate the actual recurrence rate. The last major limitation of this study was the lack of adequately large amounts of data for designing AI algorithms. Inorithms. In fact, collecting this big data is very difficult and requires the cooperation of several teams from multiple centers and it would be better that several centers from several countries share their data to analyses and compare together via traditional and AI methods.

## Ethics approval and consent to participate

The current study was approved by the ethic committee of Tehran heart center, Tehran University of medical sciences with NO: IR. TUMS.THC.1402.064 and all protocols of ethic were applied.

## Consent for publication

Not Applicable.

## Patient consent

Written informed consent was obtained from the patient for publication and any accompanying images. A copy of the written consent is available for review by the Editor-in-Chief of this journal on request.

## Availability of data and materials

The datasets used and analyzed during the current study available from the corresponding author on reasonable request.

## Funding

Not Applicable.

## Authors' contributions

EMN, SS, AVF, AYS, FMS, HBK, AZ and AB participated in the planning of project, study, analysis of data, and writing.

## Declaration of competing interest

The authors declare that they have no known competing financial interests or personal relationships that could have appeared to influence the work reported in this paper.
